# An empirical analysis of the relationship among price, demand and CO_2_ emissions in the Spanish electricity market

**DOI:** 10.1016/j.heliyon.2024.e25838

**Published:** 2024-02-08

**Authors:** José María Luna-Romera, Manuel Carranza-García, Ángel Arcos-Vargas, José C. Riquelme-Santos

**Affiliations:** a Department of Computer Languages and Systems, ETSII, University of Seville, Seville, 41012, Spain; b Department of Industrial Organization and Business Management I, ETSI, University of Seville, Seville, 41092, Spain

**Keywords:** Energy generation, $CO_{2}$ emissions, Clustering, Machine learning

## Abstract

$CO_{2}$ emissions play a crucial role in international politics. Countries enter into agreements to reduce the amount of pollution emitted into the atmosphere. Energy generation is one of the main contributors to pollution and is generally considered the main cause of climate change. Despite the interest in reducing $CO_{2}$ emissions, few studies have focused on investigating energy pricing technologies. This article analyzes the technologies used to meet the demand for electricity from 2016 to 2021. The analysis is based on data provided by the Spanish Electricity System regulator, using statistical and clustering techniques. The objective is to establish the relationship between the level of pollution of electricity generation technologies and the hourly price and demand. Overall, the results suggest that there are two distinct periods with respect to the technologies used in the studied years, with a trend toward the use of cleaner technologies and a decrease in power generation using fossil fuels. It is also surprising that in the years 2016 to 2018, the most polluting technologies offered the cheapest prices.

## 1 Introduction

Since its commercial operation in the last years of the nineteenth century, electricity has played an important role in the economic development of society. Citizens' concerns have evolved from the search for sufficient and low-cost electricity, to the impact it can have on the environment [1], the Paris Agreement [2] and the European Union Emissions Market [3], or problems in supply security, recently exacerbated by recent conflicts in supplier countries [4].

These new societal aspirations were formalized with the definition of the Energy Trilemma [5], in which cost, sustainability, and supply security are explicitly stated as the most essential aspirations of society. While society's concern until then had focused on producing energy in sufficient quantity and at low cost, with the Trilemma two new concerns have emerged: Sustainability and supply security.

Technological progress has contributed to the simultaneous achievement of the three aspirations of the Trilemma (cost, security, and sustainability) through cost reductions and efficiency gains of renewable and storage technologies [6]. As an illustration, it can be observed that the costs of photovoltaic and storage technologies have been reduced by more than 10 times in the last 10 years. In addition, the fact of internalizing in many states the negative externalities caused by some generation technologies through a $CO_{2}$ market, as is the case in Europe with the establishment of the greenhouse gas emissions trading scheme in 96/61/C Electrification of the economy is one of the keys to progress in the three coordinates of the Trilemma, so the performance of this market is key to progress on the energy transition.

It should be noted that, in the Spanish electricity market, as in all other liberalized countries, the technology that sets the price of energy is extremely important, since the price at which energy is consumed will depend on it. Therefore, it is interesting to perform an in-depth analysis of technologies that have been used in recent years, as well as the relationships associated with $CO_{2}$ emissions. One of the techniques that exists to conduct an in-depth analysis of these relationships is Machine Learning (ML) [7], [8], [9], [10]. ML is seen as a part of artificial intelligence and one of its purposes is to extract knowledge from data. ML is also one of the most widely used techniques for knowledge discovery, and its use can be found in many areas of knowledge such as chemistry [11], [12], computer vision [13], [14], [15] or data streaming [16], [17], [18]. Furthermore, we can find numerous studies that use ML to predict electricity consumption [19], [20] or price prediction [21], [22]. However, to the best of our knowledge, there are no studies in the literature that describe the technology that sets the price of energy, as well as the correlation between it and the final price of energy.

In this article, an analysis of technologies that set the price of electricity for 6 years (2016-2021) has been carried out. The analysis consists of two different parts. First, unsupervised learning techniques have been applied to search for relationships between the technological profiles, as well as to characterize them. Moreover, a study of the correlation between marginal technologies and the price of energy has been carried out. In summary, this paper presents three major contributions to the Spanish electricity market:

1. Introducing a novel methodology: This paper presents a pioneering methodology that uncovers new information on energy generation technologies used to determine energy prices. Using descriptive statistics, clustering, and statistical inference, the study identifies similarities among various time series data related to electricity technologies and their impact on energy prices in Spain.
2. Different periods of analysis: The study reveals two distinct periods within the 6-year analysis period (2016-2021). The first period (2016-2018) is characterized as the most polluting, where less clean technologies played a significant role in the determination of energy prices. During this period, lower demand hours, such as overnight and weekends, aligned with higher prices influenced by the most polluting technologies. In contrast, the second period (2019-2021) represents the cleanest phase, dominated by green technologies as the primary source of energy generation.
3. Shift towards cleaner technologies: The results obtained from the clustering analysis indicate that all clusters in the second period have centroids below the levels observed in the previous period. This finding signifies a significant transition toward cleaner energy technologies, as evidenced by lower centroid values. This shift highlights the increasing reliance on green technologies and their positive impact on reducing pollution and improving the overall energy landscape.

In conclusion, this study offers a fresh perspective on energy generation technologies and their role in shaping energy prices. By analyzing real-world data using advanced statistical techniques, we gain valuable insight into the transition towards cleaner and more sustainable energy sources. These findings have practical implications for policy makers and industry stakeholders in navigating the evolving energy market landscape. In this line, regulators can use the results obtained to perform an evaluation of public energy policies, monitoring the impact of these policies on prices and emissions over the period considered. It can also help power plant owners determine their hourly bids to participate in the electricity market, providing information on their behavior during the week, the hour and their evolution over time.

The rest of the paper is organized as follows: Section 2 details the different agents involved in the Spanish electricity market from the generation of energy until it reaches the end customer, including how the energy price is calculated. Section 3 presents related works that contribute to the state-of-the-art in this topic. Section 4 shows the methodology followed, as well as the materials that have been used for the experiments. Section 5 presents the results of the experimentation, and Section 6 the discussion of them. And finally, the conclusions and future work are exposed in Section 7.

## 2 Spanish electricity market

**Figure 1:**
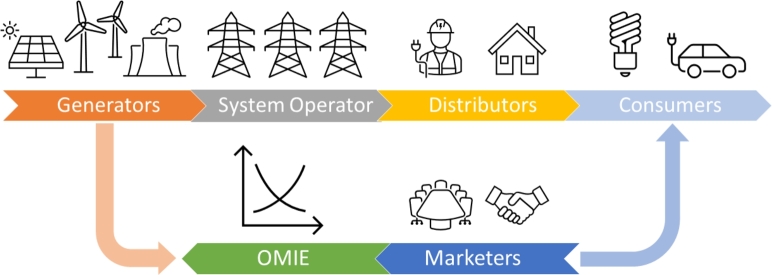
Entities involved in the electricity system.

In the current Spanish electricity system, there are several agents responsible for bringing the energy to the final destination. Fig. 1 represents the relationships and how the agents interact between them. The generators, who are the organizations that produce electricity; the System Operator, who regulates the functioning and operation of the transmission grid; distributors, who carry the electricity from the high voltage transmission grid to the low voltage socket at home; and finally, the marketers, who are the intermediaries between the entire electricity grid and the consumers.

**Figure 2:**
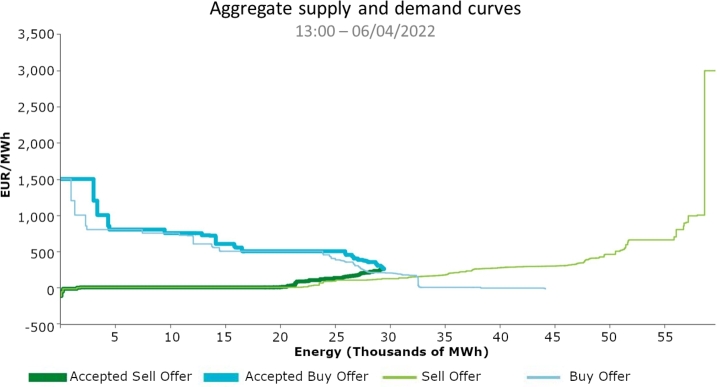
Price Curves for Buyers and Sellers at 1 PM on April 6, 2022.

The electricity market has a regulatory body, which is OMIE, and establishes legal mechanisms so that buyers and sellers can buy and sell energy [23]. That is, OMIE tries to bring the distributors, who purchase the energy, and the generators, which generate the energy, into an agreement. On the one hand, suppliers demand to the OMIE a sufficiently high price to guarantee its entry into the market. It is as if, in fact, their demand would have a rigid short-term performance. On the other hand, generators offer the minimum price they wish to sell their energy, which according to economic theory should be their short-run marginal cost. Then, in a graph, all buyers are placed first in order of the price they are willing to pay, from highest to lowest. Generators are then also ordered according to the price at which they want to sell their energy, but from the lowest to the highest (Fig. 2). The graph represents these data with two curves, and the place where the two curves intersect will be the agreed price for all the power at the time on that day [24].

For both sellers and buyers to the left of the clearing point, their bids are considered to have been accepted and an agreement has been reached. All buyers with matched bids will buy power from the sellers who have also matched their bids at the agreed price. This operation is executed every hour of the day and is planned the day before. However, within each day, intraday markets exist, allowing buyers and sellers to change their bids slightly to get closer to the actual value.

Focus on buyers, since all suppliers must provide their customers at whatever price. Therefore, in the curve, we will find some industries that prefer to stop their production if the price of energy is very high, and finally we will find storage systems that are interested in buying energy only when it is very cheap.

Also, the generators are placed on the curved as follows: The first places are occupied by the lower bids (lower marginal-cost technology facilities, such as nuclear, photovoltaic, wind, some kind of hydro, and others, so they usually bid close to zero cost. Some kind of hydroelectric facilities (pumping and reservoirs) that, despite being renewable, can manage their resources and, therefore, always try to bib themselves to obtain the best possible market price. And finally, there is fossil generation, which offers a higher price due to variable fuel and emission costs. Therefore, renewables will bid for their generation at a low price, whereas fossil generation will bid for a higher price. That is, the price of energy will be directly influenced by the renewable energies in the system.

## 3 Related work

$CO_{2}$ emissions have been one of the recurring topics of discussion in recent years. Numerous works deal with the subject from different points of view [25]. The authors of [26] studied the trends in $CO_{2}$ emissions in the United States from the 1950s to today. In the study, they conclude that one of the reasons why in the last 15 years they have achieved enormous reductions in emissions has been the disuse of coal-fired generation instead of natural gas and renewable energies, such as wind power. Another aspect to consider in this case is the drop in the price of gas, which favors the use of this type of energy over others. Li et al. [27] made a decomposition analysis to study the effects of $CO_{2}$ emissions from the electricity sector. The authors claim that economic activity, population, and emission coefficient have a positive effect on increasing $CO_{2}$ emissions; however, the power sector is significantly different depending on the economic level of the country. The study states that economic activity is one of the main causes of $CO_{2}$ emissions.

To our knowledge, there is no detailed study on $CO_{2}$ reductions in Spain. However, numerous related studies can be found using data collected in China. For example, Wei et al. [28] analyzed the use of different technologies over time, as well as the $CO_{2}$ emission of these technologies in China. Estimation and decomposition methods reveal that renewable energy has great promise in reducing emissions. Renewable energy generation is expected to replace coal as China's main energy source for power generation in the future due to large-scale development, technological progress, and cost reductions. Furthermore, Zeraibi et al. [29] investigated the drivers of the carbon footprint in the BRICS nations (Brazil, Russia, India, China and South Africa) between 2003 and 2018, revealing long-term cointegration links. The analysis highlights that urbanization, greenfield investments, and economic complexity hinder and encourage the transition to renewable energy, necessitating an active role for the industrial sector in enhancing economic complexity and increasing investment in clean and renewable energy sources for the BRICS countries. A related article on $CO_{2}$ emission reduction can also be found here [30]. This paper focuses on China's efforts to reduce carbon emissions since the introduction of the “double carbon” target in 2020. The study specifically examines the energy and heating sector, which represents approximately 50% of China's total energy consumption. Using input-output investigation and various analytical methods, the research analyzes the reduction of structural emissions in China's power and heating industry from 2007 to 2015. The findings reveal that the minimization of CO2e in this sector is significantly influenced by energy intensity, input composition, and energy structure. The study emphasizes the importance of using low-carbon energy sources to achieve effective emission reduction in the power and heating sector. The results of the research offer valuable information to formulate strategies to control $CO_{2}$ growth and accurately reduce emissions in the electric heating industry.

On the other hand, Jahanger et al. [31] examined the implications of achieving carbon neutrality in the main manufacturing countries, such as China, the United States or the United Kingdom, using the moment quantile regression method for the period 1990-2020. The results demonstrate the effectiveness of energy efficiency and renewable energy in reducing greenhouse gas emissions, and the manufacturing sector has the potential to offset its emissions through improved energy efficiency. Additionally, the validity of the Environmental Kuznets Curve hypothesis is confirmed in these countries. Furthermore, Kim [32] showed the capital investment that would have to be made to reduce $CO_{2}$ emissions by substituting fossil generation for nuclear and renewable energy. Furthermore, they demonstrate that nuclear power generation may be more efficient than renewable energy generation in terms of global emission. The goal of Yan et al. [33] was to study the effect of low-carbon innovation on $CO_{2}$ emissions from the perspective of heterogeneous classifications in the technological domain. Low-carbon innovation has no major influence on $CO_{2}$ emissions, according to empirical findings. Mai et al. [34] introduced and applied a new method to determine the cost reduction requirements for zero-emission energy generation systems to achieve a targeted and significant adoption. They calculated the levelized cost of energy that each technology must achieve by 2050 to allow the United States to achieve national incremental penetrations of up to 15%. The methods used evaluate the multiple and changing costs and values of various generation alternatives considering their distinct characteristics.

On the other hand, there are several works that present new studies on the electricity market applying machine learning techniques. There is a novel method for predicting short-term bus loads using a clustering algorithm [35]. The authors propose three forecasting models by combining the load profile given by clustering and obtaining promising results. There is another publication in which clustering techniques are used to improve the prediction of electrical load [36]. Johnpaul et al. [37] published a work in which they propose a probabilistic representational learning method to group time-series data with energy data. The identification of the environmental consequences of marginal electrical supplies in consequential life cycle assessments is discussed by Lund et al. [38]. The methodology used includes defining a relevant energy system and selecting an energy system analysis model and a life-cycle assessment tool. It shows statistical data, such as differences in electricity generation between summer and winter, day and night, for a single hour. Furthermore, Amri et al. [20] group customer data using the K-Means algorithm to process electricity usage data, including power data in kWh meters. The goal of this grouping is to determine similar patterns in the use of client electricity. The study reveals promising results, such as the use of electricity over time and the fact that most customers are classified into the lowest electricity consumption cluster.

The marginal cost of energy production after accounting for fixed outages is calculated using density estimation [39]. In this paper, the adoption of a density estimate approach to smoothing LDC to calculate marginal costs in real time has been demonstrated using a case study on the Maharashtra power system. The essential mathematical theory of continuous-time marginal power pricing is presented by Parvania and Khatami [40]. The continuous-time unit commitment problem is first formulated as a constrained variational problem, and then the continuous-time economic dispatch problem is defined, with the binary commitment variables locked at their optimal values. The numerical results show that the continuous-time marginal price reflects the behavior of power systems with constantly changing load and generation schedules.

## 4 Method and materials

In this section, the datasets that were used in the study and the proposed methodology are presented. First, the content and characteristics of the datasets are analyzed, and the construction of the dataset is also detailed (Section 4.1). Finally, we describe the practical framework step by step with the procedure that was followed in the following sections.

### 4.1 Electrical technologies dataset

In this study, our starting point was a downloaded data set that contained information on electrical technologies that influence the prices of the hourly market. These data were sourced from ESIOS [41], the Spanish electrical network operator responsible for information and management tasks related specifically to the electricity market. The dataset encompasses information spanning from June 1, 2016, to June 17, 2021 (a total of 4 years). We chose this timeframe due to a significant event in Spain, the introduction of the “Dynamic Tariff” system. This innovative model, implemented during the specified period, is grounded in the dynamics of energy demand and supply in the market, automatically adjusting prices every hour. Consequently, prices can fluctuate according to real-time demand and energy availability.

The underlying rationale for adopting this new tariff model is to improve energy efficiency and promote electricity usage during periods of lower demand. By incentivizing consumption at such times, the aim is to reduce the overall strain on the electricity system, thereby mitigating the risk of blackouts.

**Figure 3:**
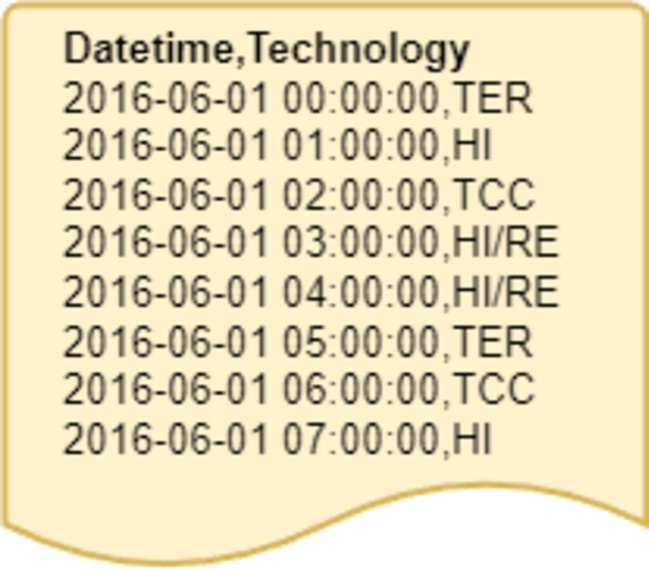
Raw data extracted from ESIOS.

The complete dataset comprises a total of 44,232 entries, exclusively containing date and time information, coupled with the technology data that dictates the energy price at that specific time, as depicted in Fig. 3. It is crucial to note that this dataset consists of categorical data, representing a time series with a label corresponding to each hour. To facilitate a comprehensive analysis, three distinct datasets have been generated from these data.

**Figure 4:**
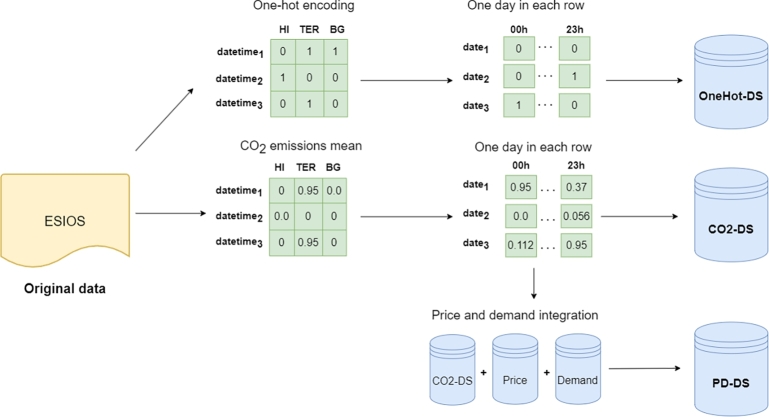
Formation of the three datasets derived from the original dataset.

Fig. 4 visually outlines the various transformations applied to the original data to derive the three datasets utilized in our analysis. The construction details for each dataset are elucidated below.

**Figure 5:**
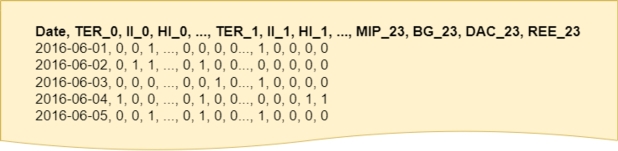
OneHot-DS representation. Features are summarized for space saving.

The first dataset is a time series whose values are generated through one-hot encoding of the utilized technology (*OneHot-DS*). This encoding is constructed based on the technologies that have been active over time. Specifically, if hydro generation sets the price at a certain time, its corresponding value will be one, while the values for the remaining technologies will be zero. On the contrary, if multiple technologies set the price during a time slot, the result of the one-hot encoding will be all those technologies set to 1. This dataset encompasses the one-hot encoding values for all hours (organized by columns) across days (organized by rows) over the four-year data span. The objective is to conduct a clustering analysis by days and analyze the centroids by hours of the day, as discussed in later sections. Fig. 5 visually represents the structure of the data in this dataset.

As illustrated, the first row contains column names in bold, representing the timestamp at the beginning, followed by the names of each technology along with the corresponding time. Here, time 0 refers to midnight, and time 23 corresponds to 11 PM. In other words, the data corresponding to the attribute “$TER_{0}$” will be 1 if thermal generation has set the energy price during that hour. The abbreviations for the technologies can be found in Table 1.

**Table 1:** Mapping of technology to tons of equivalent CO_2_ emissions, sorted by CO_2_ emission levels. Source: REE [42].

Technology	Acronym	$tCO_{2}$eq/MWh
Hydro generation	HI	0.000
Pumping storage generation	BG	0.000
Supply contract REE-EDF	REE	0.068
Renewable, Co-generation and wastes	RE	0.112
Imports	II	0.140
Reseller (sale)	CO	0.168
Surplus forward market	DAC	0.168
MIBEL import from Portuguese system	MIP	0.168
Combined cycle thermal generation	TCC	0.370
Conventional thermal generation	TER	0.950

**Figure 6:**
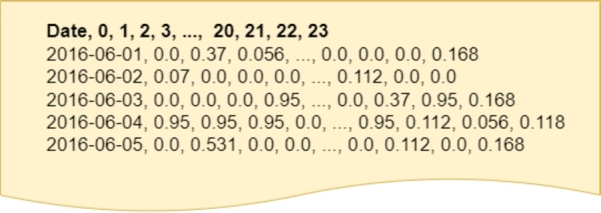
CO2-DS representation. Features are summarized for space saving.

On the other hand, the second dataset with which we have worked is made up of the $CO_{2}$ emissions that the use of each marginal technology entails (*CO2-DS*). In this case, the information on $CO_{2}$ emissions derived from each technology has been integrated. Instead of applying the one-hot encoding as in the first dataset, in this case, the $CO_{2}$ emissions of each technology have been used and replaced. Therefore, if there is more than one technology that marks the price in an hour, the label is replaced by the arithmetic mean of the emissions of those technologies. The shape of this dataset is the same as the *OneHot-DS*, that is to say, each instance represents a time series with the $CO_{2}$ emission of each hour (by columns) on each day (rows). Fig. 6 shows an example of how the data would look in the dataset. As can be seen, CO2-DS contains only one column for each hour, and the value represented by that column is the average of the $CO_{2}$ emissions of the technologies that have set the price at that hour. For example, on 2016-06-01, at midnight, the average emission value of energy pricing technologies was 0.0 $tCO_{2}$eq/MWh, while at 1 am the emission was 0.37 $tCO_{2}$eq/MWh.

In order to construct *CO2-DS*, we utilized the average $CO_{2}$ emission levels associated with each technology, as detailed in Table 1 [42], [43], [44]. This table includes all the energy sources involved in our study, including hydroelectric generation (HI) and pump storage generation (BG), both of which exhibit zero $CO_{2}$ emissions. Additionally, it incorporates other technologies, such as the import of the MIBEL Portuguese power system (MIP) with an emission level of 0.168. Furthermore, we considered technologies such as combined cycle thermal plants (TCC) and conventional thermal plants (TER), which exhibit the highest emissions at 0.370 and 0.950, respectively.

Finally, the third dataset (*PD-DS*) is used to study the correlation between electricity price and demand and mean emissions from marginal technologies. This dataset is the result of combining the $CO_{2}$ emission dataset (*CO2-DS*) with two other datasets that include the price of electricity and the demand for electricity by hour. The result of combining these three datasets is a new one in which we can find $CO_{2}$ emissions, price and demand by day and by hours. In this way, correlation analysis can be studied.

Data analysis has been carried out from three different points of view: a statistical analysis, in which a visual of the composition of the data is made from a purely statistical point of view (Section 4.2); a clustering analysis, in which similarities are sought between technologies that have occurred over the days (Section 4.3); and finally, based on the results obtained in the two previous analyses, we have measured the correlation between the price of electricity and the demand (Section 4.4).

### 4.2 Statistical analysis

First, a statistical analysis of the data was performed. Calculations such as mean, median, variance, standard deviation, chi-square, and interval counters are performed to extract information to help us understand the meaning of the data received. These calculations have been made separated by year, month, week, day of year, day of the month, day of the week, by hours and by consumption rate brackets, taking into account days off and holidays.

### 4.3 Clustering analysis

In the second stage, clustering analysis has been carried out to look for similarities between the technologies that have defined the time series daily. The k-means clustering algorithm is used to group the different data entries into groups of similar characteristics to help us understand and categorize these entries [45]. K-means was chosen because it is one of the most widely used clustering algorithms in the literature [46], [47], [48]. The analysis has been carried out on both datasets, that is, both the time series dataset composed of price-setting technologies (*OneHot-DS*) and the dataset constructed on the basis of $CO_{2}$ emissions (*CO2-DS*). To decide on the optimal number of clusters (*k*) to apply, we have followed the method that was applied by Luna-Romera et al. [49], [50], so that two internal cluster validation indices have been used: Davies-Bouldin [51] and Silhouette [52]. Once the optimal *k* study has been carried out, the K-means was applied and the resulting clusters have been characterized, the results were incorporated with the year, month, week, day of the year, day of the month, day of the week, and hours, in the same way as described in Section 4.2.

### 4.4 Correlation with price and demand

As mentioned in Section 4, *PD-DS* was built with the aim of studying the correlation between the price of electricity and demand. This dataset has been used to cross-check the information to analyze the correlation between both time series. Linear, Pearson, and Spearman correlations were used to determine if there is a correlation between marginal technology and price and between marginal technology and demand. Finally, an interval technology counter was used to determine for each interval the number of entries that were above or below the mean for both price and demand, applying the chi-square to the results.

## 5 Results

This section describes the results obtained from the methodology detailed in Section 4. This section follows the same structure as the previous section: first, the results of the statistical analysis, then the results of the clustering, and finally the correlation between marginal technology and price.

### 5.1 Statistical analysis

In this section, we first studied the distribution by years of technologies based on their $CO_{2}$ emissions. Table 2 shows the $CO_{2}$ values grouped in intervals of amplitude 0.1 from 0 to 1. As can be seen, in 2016, 45.95% of the data belonged to the interval whose $CO_{2}$ emissions were the lowest. Furthermore, the second interval had 17.87% and 22.47% to the interval whose technologies had the highest $CO_{2}$ emissions. Furthermore, we can observe that there is a growing trend in the use of technologies with low $CO_{2}$ emissions, and the use of more polluting technologies decreases over time, from 22.47% in 2016 to 2.60% in 2021.

**Table 2:** Percentage distribution across CO_2_ intervals by year.

Interval	2016	2017	2018	2019	2020	2021
[0.0, 0.1)	45.95	50.38	63.12	43.45	51.86	66.22
[0.1, 0.2)	17.87	15.32	14.91	22.37	25.79	23.24
[0.2, 0.3)	1.54	0.74	0.23	1.66	0.80	0.15
[0.3, 0.4)	9.75	12.02	4.93	23.32	18.61	7.69
[0.4, 0.5)	0.88	0.58	0.21	0.29	0.09	0.07
[0.5, 0.6)	1.29	0.67	0.17	0.25	0.13	0.00
[0.6, 0.7)	0.25	0.29	0.14	0.30	0.06	0.02
[0.7, 0.8)	0.00	0.00	0.00	0.00	0.00	0.00
[0.8, 0.9)	0.00	0.00	0.00	0.00	0.00	0.00
[0.9, 1.0]	22.47	20.00	16.30	8.37	2.68	2.60

It should be noted that most of the values are centered on 4 intervals; for that reason, the table has been simplified and summarized by omitting the less frequent intervals (less than 2% of the total). Therefore, Table 3 has been organized into the following 4 groups: **A** [0, 0.1) which are mainly BG and HI; **B** [0.1, 0.2) which is mainly RE; **C** [0.3, 0.4) which corresponds to TCC; and **D** [0.9, 1] which is mainly TER. Since these four groups have been established, it should be noted that the groups are arranged in order of contamination, so that group A is the group that pollutes the least, while group D is the one that pollutes the most. From this point on, only these 4 intervals [A-D] will be referred to in order to simplify the notation.

**Table 3:** Annual evolution of the distribution percentages of total hours throughout the year across intervals.

Year	A	B	C	D	Total
2016	45	18	10	22	95
2017	50	15	12	20	97
2018	63	15	5	16	99
2019	43	22	23	8	96
2020	52	26	19	3	100
2021	66	23	8	3	100

#### 5.1.1 Analyses based on temporal data

**Figure 7:**
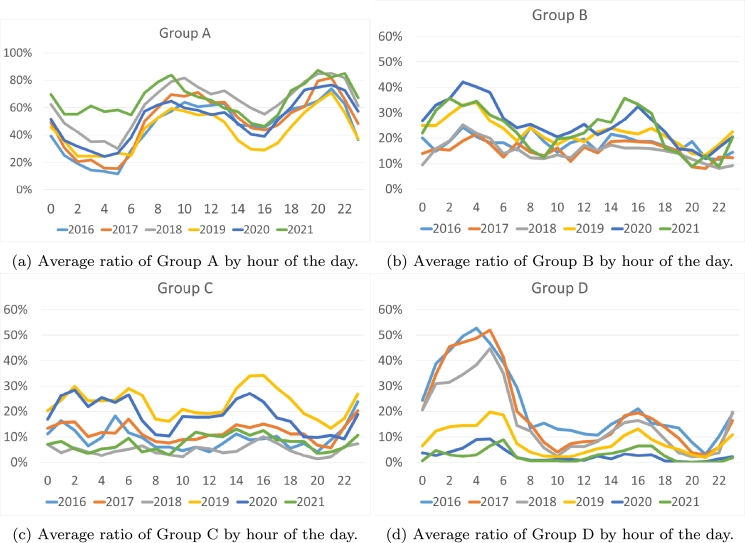
Average ratio of the pollution groups by hour of the day.

Next, we will analyze the technologies based on the time the price is established (Fig. 7). We have observed that during the years 2016-2019 there exists an increase in the use of the most polluting technologies (Group D, Fig. 7d) at night (between 1 am and 6 am). This increase assumes twice the annual average. However, the use of these technologies is considerably reduced between 8 and 10 at night. Furthermore, between 10 am and 1 pm, and between 6 pm and 10 pm, there is an increase that can reach 20% above the average of nonzero emission technologies (Group A, Fig. 7a), between 9 pm and 10 pm above 80%. On the other hand, we can observe that between 2019-2021 there is a tendency to use technologies that have a medium $CO_{2}$ emission (Groups B and C, Figs. 7b and 7c, respectively) during night and midday hours, while the use of the most polluting technologies decreases drastically during night.

**Figure 8:**
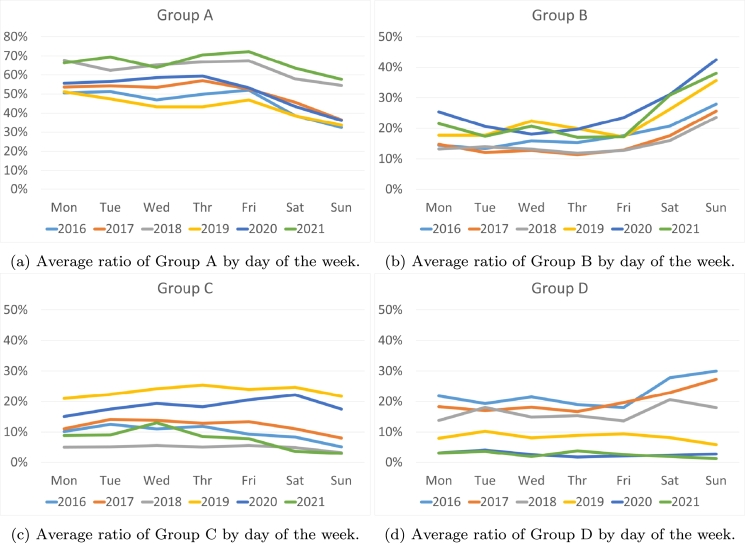
Average ratio of the pollution groups by day of the week.

Taking into account the day of the week (Fig. 8), we can observe that in 2016, weekends are much more polluting (Fig. 8d), with the most polluting technologies increasing to more than 30% and the cleanest technologies decreasing to 33%. This trend continues in 2017 and 2018 but begins to fade. However, in the years 2019-2020, medium-pollution technologies (Figs. 8b and 8c) have become prominent on weekends, doubling compared to weekdays, replacing clean technologies that decrease on weekends (Fig. 8a). While examining the day of the week, it becomes apparent that there is no discernible pattern.

**Figure 9:**
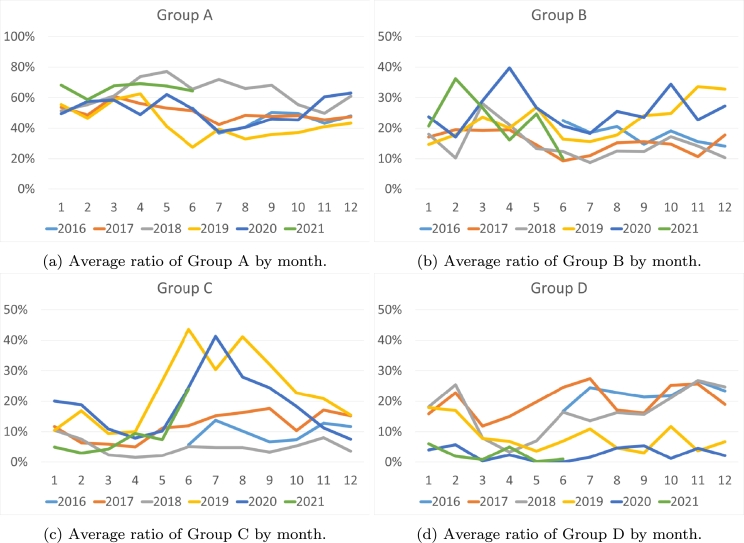
Average ratio of the pollution groups by month.

If we focus on the weeks or months of the year (Fig. 9), it becomes apparent that the differences between the months in the various years studied are not very decisive. It can be argued that the summer weeks are generally more polluting, marked by a notable decrease in the use of clean technologies (Figs. 9a and 9b) and an increase in other sources (Figs. 9c and 9d). This outcome aligns with expectations, as there is minimal generation of hydro and wind power during the summer months. In addition, there is a notable increase in polluting intervals during the weeks of November and early December.

From this first frequency analysis, three conclusions can be drawn:

• The least polluting marginal technologies have increased from 63% in 2016 to 89% in 2021. On the contrary, the most polluting have decreased from 22.4% to 2.6%. The renewable segment has grown from 17% to 23%.
• In the period from 2016 to 2018, the hours at night and on weekends had the highest presence of polluting marginal technologies, coinciding with the hours of lowest demand and lowest prices.
• This contradiction between cheap prices and polluting energy has been corrected in the second analyzed period. Between 2019 and 2021, the most polluting technologies have been used during times of higher demand (lunch and dinner hours in Spain) or during the summer months when there is higher demand due to heat and less hydroelectric or wind generation.

Therefore, it can be concluded that in the first three-year period of the study, the electricity marking the price was much more polluting than during the last few years. This was more evident in the early morning hours and on weekends. This implies a contradiction, since during the hours of lower demand and therefore lower prices, it was the most polluting energy that set the price. Moreover, in the second three-year period, clean energies set the price primarily, although there is still a certain tendency, above 20%, in the early morning and early afternoon hours to group C energies.

In the following sections, we will try to ratify or rectify these first statements of the statistical study using two complementary approaches. First, an unsupervised approach applies clustering to the days according to the marginal technology pollution of their 24 hours. The second approach will be carried out by means of a statistical analysis that will provide us with information on whether the distribution of demand values and prices is independent of the marginal technology.

### 5.2 Clustering

This section presents the results of the clustering analysis conducted on *OneHot-DS*, as described in Section 4.1. Given the observed trend of change within the 2016-2021 interval, we opted to divide the study into two subintervals to draw more precise conclusions. Consequently, the clustering algorithm was applied separately to the 2016-2018 and 2019-2021 intervals.

For each clustering analysis, we will showcase the centroids, representing the average values of the elements belonging to each cluster. Specifically, these centroids denote the average $CO_{2}$ emission for each hour. Furthermore, a summary table will outline the most significant characteristics of each cluster. To provide a complete view of the evolution of the cluster, a Sankey graph will be presented, illustrating changes as the value of *k* increases.

As highlighted earlier, all clustering analyzes involved a preliminary study to determine the optimal number of clusters [49], [50]. Silhouette and Davies-Bouldin indices were employed for this purpose. The results of these indices indicated that the optimal number of clusters falls within the range of 3 to 5. Consequently, for experimentation, clustering solutions were explored using *k* values of 3, 4, and 5, allowing us to examine the evolution of the clusters.

#### 5.2.1 *OneHot-DS* clustering for 2016-2018

**Figure 10:**
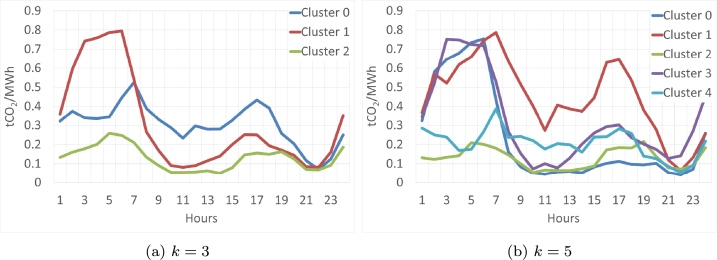
Centroids of *OneHot-DS* for various clustering solutions with each *k* value in the 2016-2018 period.

Fig. 10 displays the clustering centroids for solutions with k=3 and k=5. It is important to note that clustering was applied to the database organized by days, representing the 24-hour time series. As depicted in Fig. 10a, for k=3, three distinct clusters emerge: Cluster 1 exhibits high emission levels during the early hours of the day, with emissions dropping to lower levels for the remainder of the day; Clusters 0 and 2 share similar shapes, but Cluster 0 has higher emissions than Cluster 2.

In contrast, the centroids for k=5, illustrated in Fig. 10b, reveal two types of clusters: those with high emissions during the early hours and low emissions for the rest of the day, as seen in Clusters 1, 0, and 3; and Clusters 2 and 4, which experience some variations in emissions throughout the day, but generally maintain low levels. To summarize the centroids, the most significant characteristics of each have been consolidated in Table 4.

**Table 4:** Percentage of elements in each cluster and a summary of the elements for the 2016-2018 period.

k	3	4	5
Cluster 0	29% Low values in the early hours. Peaks at 6 and 16	27% High values in the early morning Peak at 3 pm-4 pm	26% High values in the early morning Low values

Cluster 1	45% High values in the early morning Low values the rest of the day.	22% Average values Peak at 6 am	15% High values in the early morning. Peak at 6 am and 3 pm-4 pm Medium to high values in the morning and afternoon.

Cluster 2	26% Low values in the early hours. Low values.	31% High values in the early morning Low values elsewhere.	20% Low values

Cluster 3	—	20% Low values.	22% High values in the early morning.

Cluster 4	—	—	17% Low values Peak at 6 am

Table 4 shows the different clustering solutions by columns (k=3, k=4, and k=5), and clusters by rows. It must be remembered that each solution cluster of each *k* is different and independent, that is, cluster 0 of k=3 is not related to cluster 0 of k=4. This analysis of the equivalences of the clusters and their evolution will be carried out. Focusing on the results of the table, it can be observed that almost half of the days, the most polluting technologies set the price for the early morning hours. And around 25% of the days clean electricity was a marginal technology.

**Figure 11:**
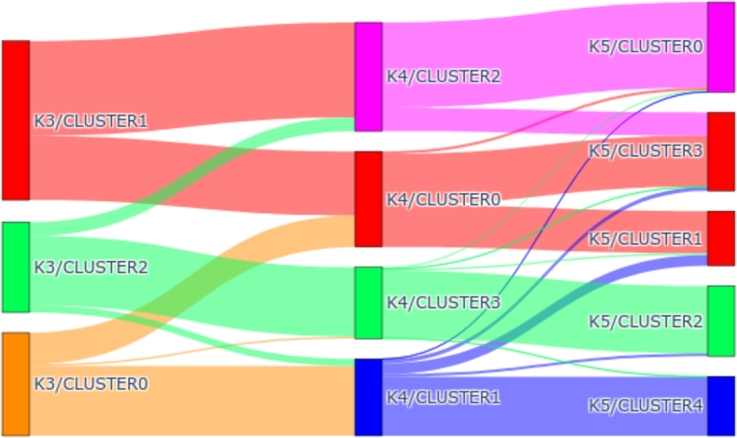
Sankey diagram depicting the transfer of elements as the *k* value changes during the 2016-2018 period.

As mentioned above, the clustering analysis has been carried out taking 3 different *k*. The evolution of the clusters as the number of elements increases is detailed below. To do this, this transformation has been illustrated using a Sankey diagram. In this diagram, we can see how the elements are distributed among the different clusters. Fig. 11 shows how clustering evolves as we increase the number of clusters with *OneHot-DS* in the period 2016-2018. As can be observed, from k=3, while clusters 0 and 2 are mainly related to clusters 3 and 1 in k=4, respectively, cluster 1 is divided into clusters 2 and 0. The next evolution, from k=4 to k=5, we find a similar situation; the clusters that were mostly matched by a single cluster do so again in this new change, with clusters 3 and 1 corresponding to clusters 2 and 4, respectively. Furthermore, clusters 0 and 2 correspond to clusters 0 and 1, respectively, and also create the new cluster 3. With these data, we could confirm that clusters 0 and 2 are completely independent of the rest since, although we increase the number of clusters, the elements remain united until the end. However, cluster 1 in k=3 has undergone the most changes, having been split into two clusters in both modifications of *k* that were made.

#### 5.2.2 *OneHot-DS* clustering for 2019-2021

**Figure 12:**
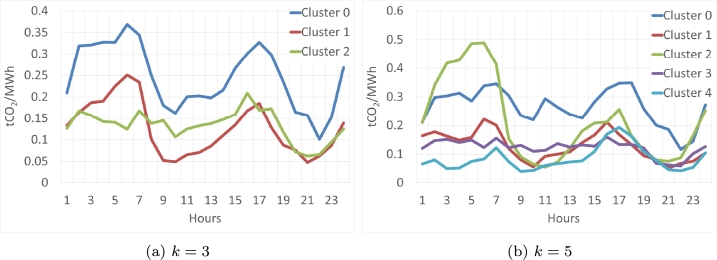
Centroids of *OneHot-DS* for various clusters with each *k* value in the 2019-2021 period.

In this section, the results of the *OneHot-DS* clustering during the second period are presented. The centroids of the clustering solution are illustrated in Fig. 12, while Figs. 12a and 12b display the centroids for k=3 and k=5, respectively. Focusing on the k=3 solution (Fig. 12a), the highest emission value corresponds to Cluster 0, reaching 0.37 at 5:00 a.m. This scenario was not observed in the previous period, where a cluster exhibited values that almost reached 0.8 with the same number of clusters. Clusters 1 and 2 exhibit similar shapes, with markedly low values throughout the day. For the centroids at k=5, we do not encounter a dissimilar situation; only Cluster 2 reaches high values, albeit not exceeding 0.5. In this instance, cluster 0 could be categorized as a medium-emission cluster, while the remaining clusters may be grouped into low-emission categories.

A summary of the main characteristics of these clustering solutions can be found in Table 5. It can be observed that the high $CO_{2}$ values are on average below 0.3 $tCO_{2}/MWh$, much lower than in the previous 3-year period. Furthermore, there are more than 30% days when the most polluting energies are priced again in the early morning. There are about 25% days with marginal polluting energies between 3 PM and 4 PM.

**Table 5:** Number of elements in each cluster and summary of elements for the period 2019-2021.

k	3	4	5
Cluster 0	30% High values in the early morning Peak at 4 pm	34% High values in the early morning Peak at 5 am and 4 pm	19% High values

Cluster 1	48% Low Values Peak at 5 am and 4 pm	25% Low values Peak at 4 pm	19% Low values

Cluster 2	22% Average values Peak at 3 pm	23% High values	23% High values in the early morning

Cluster 3	—	18% Low values	15% Low values

Cluster 4	—	—	24% Low values Peak at 4 pm

Therefore, we can conclude that, taking into account the results of the clustering analysis, the analyzed periods are completely different from each other. In the first period (2016-2018), clusters with very high emissions and others with very low ones were obtained, while in the second period (2019-2021) the clusters did not exceed emissions above 0.5, the most numerous being those whose emissions did not exceed 0.2 points.

**Figure 13:**
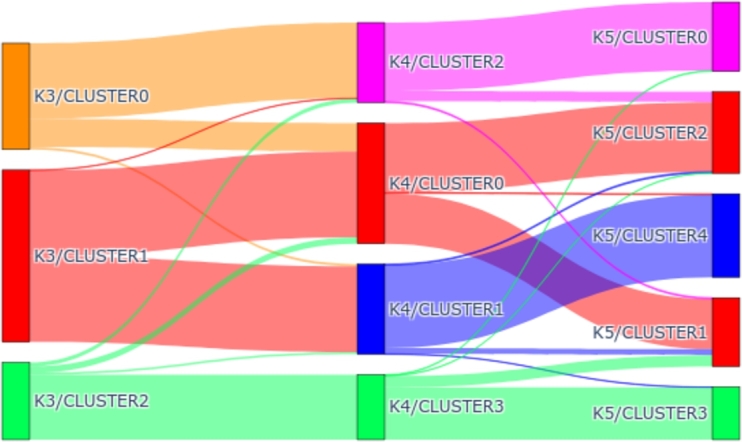
Sankey diagram illustrating the transfer of elements as the *k* value changes during the 2019-2021 period.

Fig. 13 represents it in a Sankey diagram according to the evolution of the clusters. As can be observed, cluster 2 of k=3 is directly related to cluster 3 of k=4 and k=5. On the other hand, cluster 1 from k=3 is divided into two clusters 0 and 1 in k=4, whose centroids represent high and medium emission values. We find a similar situation with cluster 0 from k=4, which is also divided into two clusters, 1 (low values) and 2 (high values in the early morning) from k=5. Most of the elements in cluster 0 in k=3 are mapped in clusters 2 and 0 of the other solutions, respectively.

### 5.3 Correlation with price and demand

This section shows the results of the different correlations. As described in Section 4.4, linear, Pearson, and Spearman correlations were used to check if there exists a relationship between marginal technology and, on the other hand, marginal technology and the final price.

**Figure 14:**
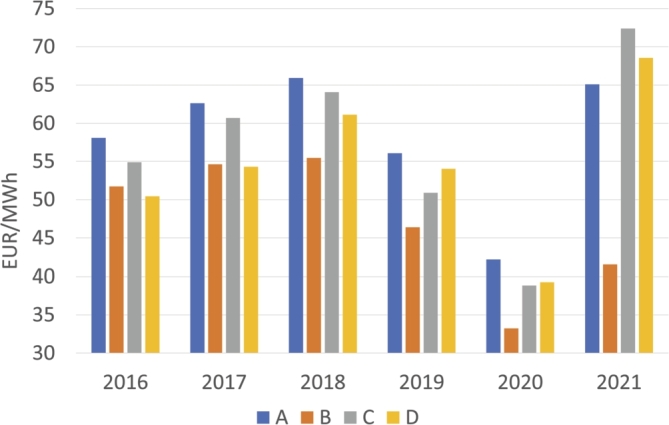
Average price values relative to marginal technologies intervals.

Fig. 14 illustrates the mean price values for the hours of each marginal technology. As can be observed, prices are higher in A technologies than in the rest in all years except in 2021 where the mean price was C and D technologies obtained higher prices. This fact indicates that cleaner technologies obtained a higher price than those that were more polluting during the first years of the study.

Focusing on the average price difference between intervals, we may observe that B and D are similar in the first years, but their difference increases later until 2021 where B is much lower than D. That is, D (the most polluting technologies) started at a price similar to that of B, but over the years it became almost twice as expensive as B.

**Figure 15:**
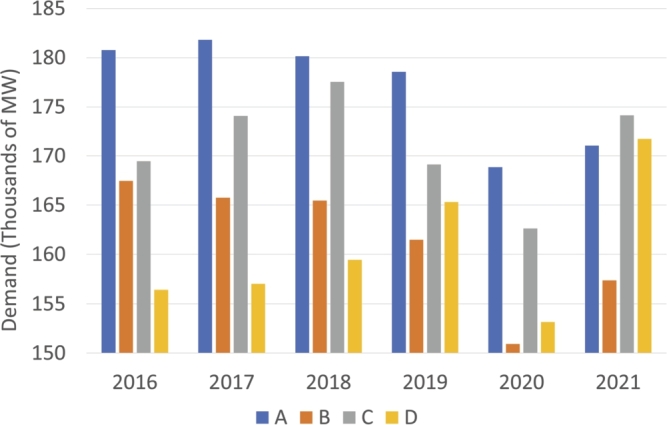
Average demand values relative to marginal technologies intervals.

On the other hand, we can observe how the demand has behaved with technology over the years in Fig. 15. As can be seen, cleaner technologies (A) have maintained their demand from 2016 to 2020, which suffered a slight decrease. At the other extreme of $CO_{2}$ emissions (D technologies), they have increased from 2016 to 2021 until they reach demand values similar to those of A. However, an interruption in 2020 can be observed due to the confinement suffered by the country due to the COVID-19 pandemic and, therefore, to the drop in demand due to the cessation of many key economic activities such as tourism. This fact could indicate that as the years go by, more polluting technologies are used to the detriment of green technologies. On the other hand, if we look at B, we can observe that its demand levels remain stable throughout all years, while C, which is used more in the first half of the study (2016-2018) and suffers a decrease in the following years, even becoming the most demanded group of technologies in 2021.

In conclusion, we can establish that during the first two years, the hours in which the marginal technology was less polluting were characterized by a higher demand and a higher price. On the other hand, the hours in which the price was set by the most polluting technology were those with the lowest price and the lowest demand. Over the years, the differences have become less pronounced, although there was still a certain difference in 2020. In the first half of 2021, the high price of the hours marked with technologies in the C range, which also corresponded to those with the highest demand, stands out.

Finally, we will perform an analysis using statistical inference models to examine the independence or lack thereof among the three factors under investigation: marginal technology, price, and demand. To apply independent inference tests, it is necessary to label the samples for establishing comparisons between groups. In this scenario, the samples will be labeled based on the average value of one of the factors and compared with the others.

For each set of hours characterized by three factors, marginal technological pollution, price, and demand, we calculate the average value of demand within a specified period. If the marginal technology pollution is independent of demand, the distribution of the number of hours above or below the average in each of the four intervals should exhibit similarity.

A chi-square test measures whether the expected distribution in the case of factor independence is similar to the actual distribution. The mean value of the demand has been taken as the average value because it is a distribution that follows a normal distribution with almost no outliers. However, the high variability of prices makes it advisable to take the median as the value that leaves 50% of the values in each interval above or below the median.

The analysis has been performed year by year, resulting in 12 chi-square analyses in Table 6, Table 7 per year for price and demand, respectively:

**Table 6:** Frequency of occurrences above and below the median within each interval concerning the price.

	2016 Price				2017 Price			
Pollution interval	A	B	C	D	A	B	C	D
Number of hours above median	1186	293	215	273	2323	401	451	351
Number of hours below median	1174	625	286	881	2075	936	600	1399

	2018 Price				2019 Price			
Pollution interval	A	B	C	D	A	B	C	D

Number of hours above median	3755	486	247	533	2662	612	858	438
Number of hours below median	1759	818	184	889	1135	1347	1172	295

	2020 Price				2021 Price			
Pollution interval	A	B	C	D	A	B	C	D

Number of hours above median	2774	682	775	105	1536	239	159	84
Number of hours below median	1771	1580	850	130	870	657	53	17

**Table 7:** Frequency of occurrences above and below the mean within each interval regarding the demand.

	2016 Demand				2017 Demand			
Pollution interval	A	B	C	D	A	B	C	D
Number of hours above average	1603	406	232	303	2882	554	555	407
Number of hours below average	757	512	269	851	1516	783	496	1343

	2018 Demand				2019 Demand			
Pollution interval	A	B	C	D	A	B	C	D

Number of hours above average	3338	485	235	353	2363	726	991	304
Number of hours below average	2176	819	196	1069	1434	1233	1039	429

	2020 Demand				2021 Demand			
Pollution interval	A	B	C	D	A	B	C	D

Number of hours above average	2615	693	776	71	1350	299	147	66
Number of hours below average	1930	1569	849	164	1056	597	65	35

All chi-square tests are highly significant with infinitesimal p-values (of the order of 10e-53 being the largest of them). Therefore, it is safe to say that both the statistical distribution of prices and the demands are not independent of the marginal technology. This could in principle be presented as an obvious result. However, what was not expected was that once again it was clear that the hours with the cheapest prices are related to the most polluting technologies, especially in the early years of this study. Let us pay attention to Table 6, for example, on the 2016 prices, while the hours above and below average for A technologies were similar (1186 vs. 1174), for D technologies they had a 1/3 ratio (273 vs. 881). This ratio worsens to 1/4 (351 vs. 1399) and only from 2019 the trend is reversed.

Regarding the demand presented in Table 7, for 2016 the hours of marginal polluting technologies have covered less hours of demand above the average (303) than below (851), which means that polluting technologies have been used in off-peak hours. This trend is maintained in the first three years, from 2019 onward, the very few hours when the technology that marked the price was polluting. However, another characteristic of the historical series that can be observed is the increase in the hours where marginal type B technologies have marked price, with an increasing disproportion between hours below and above the average. For example, in the last full year (2020), 693 hours versus 1569, a ratio of 1 to 2 that is maintained in 2021.

Finally, we have studied the relationship between the $CO_{2}$ emissions of each technology, with their average price by applying the chi-square. It should be noted that the chi-square is a measure of the discrepancy between the observed values and the expected values in a contingency table. If the chi-square value is high, it means that the observed values and the expected values are significantly different, indicating an association between the variables. In other words, a high chi-square value suggests that the relationship between the variables is not coincidental or fortuitous.

**Figure 16:**
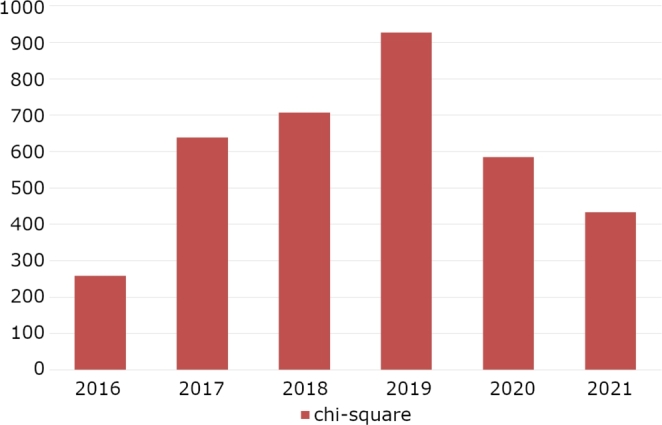
Visualization of the chi-square values between CO_2_ emissions and price. The Y-axis corresponds to the chi-square values.

Fig. 16 shows the chi-square values for all years of the study. On the other hand, Table 8 shows the results of the p-values for these statistical tests. It should be noted that the chi-square test was performed with 3 degrees of freedom. As can be seen, the p-values are below the marked significance level (alpha=0.05) and therefore the null hypothesis is rejected and it can be concluded that there is a significant association between technology and the price of energy.

**Table 8:** P-values for the chi-square analysis between technology intervals and the average price.

	2016	2017	2018	2019	2020	2021
p-value	5.32E-53	1.42E-134	2.71E-149	9.07E-197	6.71E-123	1.61E-90

## 6 Discussion

The results presented in the previous section can be summarized as follows:

(**a**) Two different periods can be distinguished in the horizon analyzed (2016-2021: A first period, 2016-2018, of higher emissions, while last years market prices are marked by cleaner technologies.
(**b**) Off-peak periods, such as at night, are the times when the most polluting technologies have set the price. This effect increases in the second period (2018-2021).
(**c**) In general, the hours of lowest demand coincide with the hours of lowest price.

**Figure 17:**
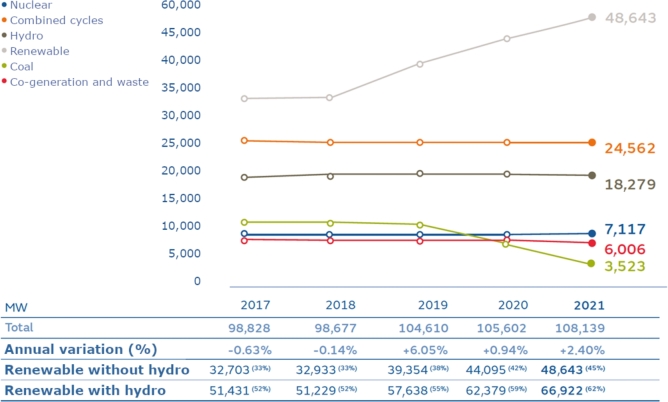
Installed power by technology in Peninsular Spain from 2018 to 2022.

The first obtained result (a) is a consequence of the structural changes implemented in the Spanish power generation system in the period considered, as a consequence of the change in the Spanish energy policy in 2018, promoted by the change of government. In this legislative mandate, the orderly closure of coal-fired power plants (with higher emissions) and the development of new renewable facilities (mainly wind and photovoltaic) were decreed, for which a program of annual capacity auctions was developed. Fig. 17 illustrates the substantial reduction in installed capacity in coal-fired power plants (depicted by the green line), that is, almost 7000 MW (equivalent to two thirds of installed capacity). At the same time, there was a notable increase in renewable generation (represented by the gray line), with a surge of almost 15,000 MW. These structural changes rationalize the identification.

**Figure 18:**
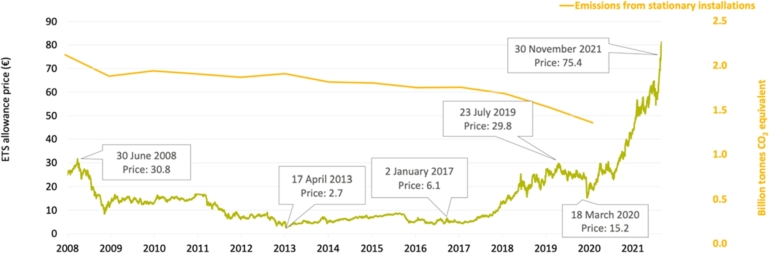
Evolution of European ETS prices and emissions produced from 2008 to 2021.

**Figure 19:**
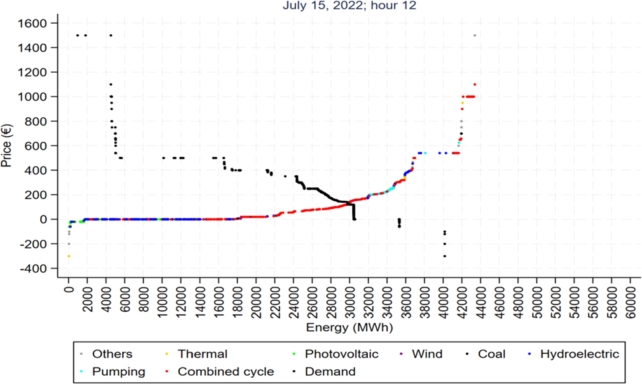
Illustrative supply and demand curves for the Iberian wholesale market on July 15, 2022, at 12:00 PM.

In addition to the reason mentioned above, we can associate the lower emissions in the second subperiod with the loss of competitiveness on the market of high-emission plants, due to the increase in emission rights and fossil fuels (mainly natural gas). Fig. 18 shows how the costs of ECTS have increased in the period under consideration, which has caused an increase in their production costs and consequently in their bids, causing them to be left out of the market several times. Fig. 19 shows the distribution of the different technologies on the supply curve [53].

Fig. 19 shows the distribution of the different technologies on the supply curve [53].

The second result (b) can also be attributed to the aforementioned change in the technological landscape of power generation. As illustrated in Fig. 17, particularly in the second subperiod (2019-2021), there has been a substantial reduction in the installed capacity of coal-fired plants, coupled with an almost 50% increase in renewables. Notably, the renewable generation installed during this period is characterized by its non-manageability, as it is contingent upon the availability of primary resources such as wind and sunlight.

Despite the significant rise in renewable generation, manageable generation, mostly fossil-based (combined cycle and coal-fired plants), continues to play a crucial role, helping to meet demand during periods of insufficient renewable resources. Given that a substantial portion of renewable generation, especially photovoltaic, is associated with daylight hours, the participation of manageable units (emitters), primarily combined cycles, explains the higher emission levels during off-peak hours, notably at night. Consequently, the higher emission levels during off-peak periods, such as nighttime, can be justified by the unavailability of solar resources during these times.

**Figure 20:**
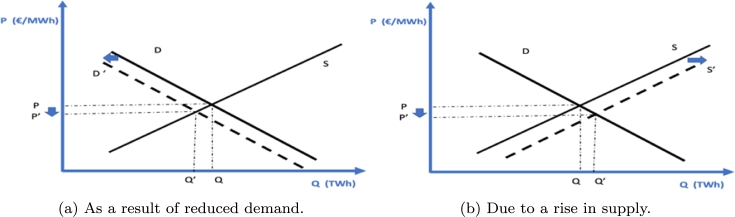
Representation of market price reduction in two different scenarios.

Lastly, the functioning of a competitive market based on marginal costs justifies the results obtained in point (c). The observation that hours of lowest demand (e.g., evenings or holidays) coincide with the lowest prices can be explained by a shift in the demand function towards the origin (depicted in Fig. 20a), leading to a reduction in prices, all else being equal (ceteris paribus). In the subsequent years, it is plausible for lower prices to manifest during peak solar production hours (central hours of the day) since the contribution of these plants would expand the supply (depicted in Fig. 20b), while the demand would remain constant (ceteris paribus). Fig. 20a illustrates the price reduction associated with a demand contraction (consistent with the model), while Fig. 20b describes the possible outcomes after the commissioning of the planned photovoltaic facilities.

Although the above points to the consistency of the results obtained, the limitations of the study can be highlighted by the fact that the emission levels adopted in the analysis correspond to those of the marginal technologies in the market, and not to the total, for which it would be necessary to gather additional information. In any case, given that the variability of the emissions will depend on marginal technology, the results obtained were found to be significant.

## 7 Conclusions and future work

In summary, this paper introduces a novel methodology to uncover new information related to energy generation technologies currently used to fix energy prices. The study uses techniques such as descriptive statistics, clustering, and statistical inference to identify similarities among various time series generated during the analysis of electricity technologies that affect the price of energy in Spain.

The results of the study suggest that there are two distinct periods within the 6-year analysis period (2016-2021). The first period, 2016-2018, can be described as the most polluting period, where less clean technologies played an important role in setting the price. The results show that hourly periods of lower demand, such as overnight and weekends, are times when the most polluting technologies have marked the price. During this initial three-year period, the hours of lower demand and lower price coincided with the most polluting technologies. The second period, from 2019-2021, can be defined as the cleanest period, where the use of green technologies is the main source of energy generation. The clustering results show that all clusters have centroids below the levels of the previous period. This conclusion is aligned with the change in energy policy implemented in Spain since 2018, in which a closure agenda for the most polluting power plants was developed. On the other hand, the evolution of allowance prices in Europe has increased from an average of 6 /t$CO_{2}$ to 60 /t$CO_{2}$ (x10), reaching peaks close to 100 /$tCO_{2}$. As a result, higher emission units (coal) suffered cost increases, which prevented their incorporation into the market.

The robust correlation observed between emissions, prices and energy demand, as analyzed in this paper, is likely to carry significant implications for the formulation of energy policy. It offers the opportunity to investigate how the capacity and technology choices for power plants, whether promoted or authorized by regulatory bodies, impact these variables, thereby influencing aspects such as inflation and environmental outcomes. However, it is crucial to approach the results obtained with caution, acknowledging the presence of numerous other exogenous parameters that can influence the outcomes, including but not limited to fuel prices (e.g., natural gas and coal) and emissions trading system (ECTS) dynamics.

While this study focused on a specific range of years, expanding the analysis to include the most recent data could reveal potential changes in trends. Such an extension would be valuable for providing insights into the contemporary energy landscape and its potential shifts. Furthermore, this work holds ecological significance by elucidating how different technologies contribute to $CO_{2}$ emissions and the intricate relationships among technology, price, and demand. Although the current study offers valuable information on the dynamics of electricity generation technologies, pollution levels, and electricity prices from 2016 to 2021, a more extensive longitudinal analysis is warranted. Future studies should consider examining data over a longer period to foster a comprehensive understanding of evolving trends and changes in electricity generation technologies and their corresponding environmental impacts.

Future studies should try to extend the period of years, review previous periods in which pollution was not taken into account, and consider the current period in which the price of electricity in Spain reaches historical records due to changes in tariffs, high inflation, and international political conflicts. It would also be interesting to apply this methodology using other clustering algorithms and approach the problem from another point of view, such as predicting $CO_{2}$ emissions. On the other hand, the relationship between electricity prices, pollution levels, and power generation technologies is multifaceted and influenced by various socioeconomic factors. To provide a comprehensive analysis, future research should consider the integration of socioeconomic data, such as GDP growth, population dynamics, and industrial activities. Incorporating these factors into the analysis can help identify the drivers of certain energy price trends and better elucidate the interaction between environmental concerns and economic realities. In addition, international politics, agreements, and regulatory frameworks play an important role in shaping a country's energy mix and environmental policies. The impact of specific policies and regulations on the price of electricity and the adoption of environmental policies should also be investigated.

## Abbreviations

• **A to D intervals**: Grouped intervals where A contains the cleanest technologies and D the most polluting technologies.
• **CO2-DS**: $CO_{2}$ emissions dataset.
• **ESIOS**: Eléctrico Sistema de Información del Operador del Sistema (Electric System Operator Information System).
• **ML**: Machine Learning
• **OMIE**: Operador del Mercado Ibérico de Energía (Iberian Energy Market Operator).
• **OneHot-DS**: One Hot Encoding dataset.
• **PD-DS**: Price and Demand dataset.

## CRediT authorship contribution statement

**José María Luna-Romera:** Writing – review & editing, Writing – original draft, Software, Methodology, Investigation, Formal analysis, Data curation, Conceptualization. **Manuel Carranza-García:** Writing – original draft, Software, Resources, Methodology. **Ángel Arcos-Vargas:** Writing – review & editing, Validation, Conceptualization. **José C. Riquelme-Santos:** Writing – review & editing, Supervision, Funding acquisition, Formal analysis, Conceptualization.

## Declaration of Competing Interest

The authors declare that they have no known competing financial interests or personal relationships that could have appeared to influence the work reported in this paper.

## Data Availability

The data utilized in this study were obtained from ESIOS [41] and are publicly accessible. All the necessary data required to replicate the experiments and conduct further analyses are available without any restrictions. Interested parties are invited to request access to the data by contacting the research team. Our commitment to transparency and openness in research is evident, and we actively encourage fellow researchers to utilize and build upon these data. The availability of data aligns with the principles of open access and scientific reproducibility. This statement fulfills the data disclosure requirements and signifies the dedication of the research team to sharing the data with the scientific community. For data access inquiries or additional information, please feel free to contact us.
